# Musculoskeletal First Contact Practitioners Undertaking a Higher Education Training Route—A Qualitative Exploration of Clinical Supervision Experiences

**DOI:** 10.1002/msc.70095

**Published:** 2025-04-03

**Authors:** Sue Innes, Sarah Golding, Philip Nardone, Caroline Kerry, Anthony Smith, Sarah‐Jane King, Fiona Blackman

**Affiliations:** ^1^ School of Sport Rehabilitation and Exercise Sciences University of Essex Colchester UK; ^2^ University Hospital Birmingham NHS Foundation Trust Chelmsley Wood Primary Care Centre West Midlands UK

**Keywords:** clinical mentorship, clinical supervision, first contact physiotherapist, first contact practitioner, musculoskeletal, professional identity

## Abstract

**Background:**

Musculoskeletal First Contact Practitioners (FCPs) are employed in primary care to assess patients and decrease workload for general practitioners. FCP training requirements are outlined in The Roadmap to Practice (RTP), which includes clinical supervision.

**Methods:**

In this qualitative study, 12 musculoskeletal FCPs who had completed FCP training at a specific English university participated in semi‐structured interviews that explored their clinical supervision in primary care.

**Results:**

Thematic analysis identified three themes: operational factors, the role of personal and professional identity and the dynamics of learning. Participants reported variation in support and organisation provided in their places of work and the impact on their learning; specific barriers related to employment contracts were highlighted. Participants reflected on personal and professional factors that were inconsistently addressed, including cultural competence and the relevance of neurodiversity for both patients and professionals. Positive elements were raised relating to learning dynamics, including accessing supervision from more than one individual and representatives from more than one profession. The bidirectional learning opportunities offered from clinical supervision were highlighted, including supervisors who are not musculoskeletal specialists accessing musculoskeletal expertise from their mentees and the FCPs benefitting from supervisors who have extensive experience of managing complex consultations in primary care.

**Conclusion:**

Clinical supervision experiences of FCPs in this study were typically positive. High‐quality supervision is dependent on stakeholders fully understanding the role and is optimised by multi‐professional involvement. Future studies could include evaluation of referral patterns and clinical outcomes of FCPs from varying employment frameworks.

## 1 Introduction

Musculoskeletal First Contact Practitioners (FCPs) are ‘physiotherapists who are able to assess, diagnose, treat, and discharge without medical input, and they are competent at managing the full spectrum of musculoskeletal patients’ (Stynes et al. 2020, 3). Musculoskeletal conditions constitute up to 30% of primary care consultations in England (NHS England (a), n.d.) where the FCP role supports General Practitioners (GPs) with this caseload, aiming to optimise patient care by ensuring appropriate expertise at the start of patient pathways (HEE/NHSE 2021). Studies demonstrate that FCPs provide safe, clinically effective, cost‐beneficial care with high levels of patient satisfaction (Walsh et al. 2024; Goodwin et al. 2021; Wood et al. 2022).

Health Education England (HEE) (known now as NHSEngland [NHSE]) published the Musculoskeletal FCP ‘Roadmap To Practice’ (RTP) (HEE/NHSE 2021), that defines the FCP role, entry criteria, education, training processes and required capabilities, building upon the Musculoskeletal Core Capabilities Framework (HEE/NHSE 2018). Training was initially evidenced via an independent ‘portfolio route’ or ‘taught higher education’ route; this was amended in 2024 so that higher education institutions now guide and support both routes. A study exploring experiences of FCPs navigating the ‘independent portfolio route’ concluded that the volume of evidence required and limited guidance was overwhelming for many, particularly novice FCPs who expressed preference for a higher education route (Carus et al. 2023).

Clinical supervision is detailed as an integral component of the RTP (HEE/NHSE 2021); however, challenges to accessing quality supervision have been previously identified in those undertaking the independent ‘portfolio route’ (Millington et al. 2024). This study explores the lived experiences of FCPs undergoing clinical supervision as part of the ‘taught route’ for fulfilment of the RTP. This study aims to:

- Explore elements associated with FCP clinical supervision, including logistics, clinicians involved, common areas of practice development and the impact of this training requirement.
- Investigate the facilitators and barriers to effective supervision.
- Support the development of supervision models for FCPs.

## 2 Method

A qualitative design was chosen applying an interpretivist approach underpinned by constructivism (Bryman 2021). This approach enables inductive analysis of data from a small number of participants with detailed exploration of individuals' experiences. The researcher assumes a central role by intuitively examining emerging meanings whilst looking for deeper interpretation. Researchers achieve this by engaging participants using personal knowledge and experience whilst undertaking personal reflexivity during the research process (Ayton et al. 2024; Braun and Clarke 2022).

The research team consisted of academics and physiotherapists experienced in FCP education, its governance framework and the clinical role. The interviewer (S.G.) possessed a shared academic and clinical (FCP) role and had previously completed the RTP requirements. A purposive recruitment strategy was followed; participants invited (via email) had all completed the clinical requirements of the RTP via a higher education route at the researchers' workplace. For this particular university module, eligible clinicians are fully qualified physiotherapists employed in the musculoskeletal FCP role and supported by at least 1 ‘clinical mentor’, a suitably qualified physiotherapist and/or General Practitioner (GP). Students may access support from more than one mentor. The term ‘clinical mentor’ is used for this module; other institutions may use the term ‘supervisor’.

Participants represented a breadth of experience and demographics including diverse UK geographical locations (Table 1). Twelve participants were interviewed, enabling a thorough exploration of the topic, collection of manageable data quantity and evidence of data saturation. Similar studies within the field had a comparable sample size (Bassett and Jackson 2022; Carus et al. 2023).

**TABLE 1:** Participant demographics.

Participant number	Gender	Age	Ethnicity	Years post registration (when underwent stage 2)	Months/years FCP experience (when underwent stage 2)	Physiotherapy degree (for registration) undertaken in UK	Job banding/equivalent banding (as per agenda for change)	Region (as per NHSE training hub)	Mentor profession	Number of mentors
1	F	43	White	21	2 years	Yes	8a	Mid & South Essex	Physio (FCP)	1
					6 months					
2	F	40	Asian/Asian British	12	2 years	Yes	7	Sussex	GP	1
3	F	32	Asian/Asian British	6	2 years	No	7	Mid Mersey	Physio (FCP)	1
4	F	41	White	17	2 years	Yes	8a	Birmingham and Sollihul	Physio (FCP)	2
					6 months				GP	
5	F	43	White	17	2 years	Yes	7	NHS Lothian^a^	Physio (FCP)	1
					8 months					
6	F	41	White	19	3 years	Yes	8a	Birmingham & Sollihul	Physio (ACP)	2
									GP	
7	M	33	White	8	6 months	Yes	7	Cheshire & Merseyside	Physio (ACP)	1
8	F	40	White	7	6 months	Yes	7	Somerset	Physio (FCP)	1
9	M	30	Arab	7	1 year	Yes	8a	Kent & Medway	Physio (FCP)	1
					6 months					
10	M	38	White	13	2 years	Yes	8a	Sussex	Physio (FCP)	1
					6 months					
11	F	44	White	19	3 years	Yes	8a	Hampshire	Physio (ACP)	2
12	M	39	Asian British	8	2 years	Yes	7	Staffordshire & Stoke‐on‐Trent	GP	1

^a^ NHS Lothian is in Scotland where there is equivalent to an FCP role; however, the musculoskeletal RTP and funding for FCPs under the Additional Roles scheme is only within NHS England (NHSE).

A semi‐structured interview guide to explore FCP's clinical supervision experiences was created by the research team. This design process was informed by key methodological principles including the need to ensure clarity of intent, enable participant honesty and optimise responses directly related to the research aims (Robson and McCartan 2016). As the research team included clinicians employed as FCPs, interview piloting within the team enabled question refinement, clarification of prompt usage and development of an interview topic guide (Appendix 1). Semi‐structured interviews (lasting 45–60 min) were conducted with consenting participants via the digital platform, ‘Zoom Workplace’ (Version 6.1.6). Interviews were recorded, anonymised for data analysis and reporting and transcribed. The interviewer had no previous relationship with the participants. Transcription, coding, analysis and reporting of findings were undertaken by team members (P.N. and S.I.), who hold academic and clinical roles. Two participants undertook data verification, and one minor amendment was made to maximise anonymity.

Thematic analysis was guided using Braun and Clarke's six phase approach (Braun and Clarke 2022) and the NVivo qualitative software programme (Version 14) utilised. Codes were initially generated by one researcher (P.N.) who had undertaken transcription; these were verified by a second researcher (S.I.) and participant (F.B.). Inter‐coder reliability was established, ensuring that groupings of data were consistent, illustrative of the raw data and the trustworthiness of data analysis could be evidenced (Castleberry and Nolen 2018). The analysis was inductive without prior identification of concepts or theories to test or explore. Initial coding provided evidence of data saturation as few new codes were required for the final interviews—markers of data saturation were verified by the participant checker. Secondary coding followed and then development of subcategories, categories and final themes.

Ethical approval for this study was granted by the University of Essex, School of Sport, Rehabilitation and Exercises Faculty Ethics Sub‐Committee (Reference: ETH‐2324‐0690).

## 3 Results

### 3.1 Demographics

Participant demographics are detailed in Table 1, and all possessed the minimum experience requirements (5 years post‐qualification with 3 years musculoskeletal experience) as set out by NHSE, ranging from 6 to 21 years post‐qualification experience. Participants had been employed as an FCP between 6 months and 3 years. There was an even distribution of role banding between 7 and 8a (Agenda for Change or equivalent), the recommended standard for this role (CSP 2018; HEE/NHSE 2021). Geographical locations of participants varied throughout England; one participant was based in Scotland where FCP equivalent roles exist; however, the musculoskeletal RTP and funding for FCPs under the Additional Roles Reimbursement Scheme (ARRS) only apply within England. Professional diversity was seen among mentors.

Table 2 summarises the coding framework. Three themes were identified for review and discussion: operational factors, the role of personal and professional identity, and dynamics of learning. Examples of quotations from participants have been included to support the presentation of themes.

**TABLE 2:** Data analysis: Coding framework.

Codes	Sub‐categories	Categories	Themes
Primary and secondary care interfacing	Links to secondary care	Service connections	1. Operational factors
Primary—Secondary pathways			
PCNs and other stakeholder support	Employer's understanding of process		
Wider barriers around supervision in primary care			
Time blocked out for supervision	Time	Resource availability	
Resource commitment for supervision			
Location logistics	Location		
Support from practice staff			
Who facilitated organising supervision	Accountability for organisation		
Contractual impact			
Personal cultural background	Cultural identity	Equality diversity and inclusivity (EDI)	2. Role of personal and professional identity
Patient's cultural background			
Neurodiversity of learners	Neurodiversity		
Neurodiversity of patients			
Impact on clinical identity	Professional identity	Exploration of professional identity of FCP	
Scope of practice			
Clear instructions	Direction from higher education institution (HEI)	Impact of directed learning	3. Dynamics of learning
Supervision structure			
Application of learning from taught stage 1 to stage 2	Teaching and assessment strategy		
Benefits of mentor not doing summative assessment			
Relationships: Mentor—Mentee—Colleagues	Impact of professional relationships	Influence of mentorship relationship	
Power dynamic			
Selecting your own mentor	Access to range of professions/professionals		
Mentorship not restricted to 1 person			
Poor mentorship strategies	Negative influences	Effectiveness of mentorship	
Cutting corners with mentorship			
Financial barriers			
Diverse caseload	Positive influences		
Useful feedback			
Time to reflect	Learning opportunity	Bidirectional learning	
Value to mentor			
Professional respect			
Patients' reactions	Patient acknowledgement of process		

### 3.2 Theme: Operational Factors

Participants reported that their clinical supervision was affected by factors related to the supervision environment, most notably the connections between primary and secondary care services. The category, ‘Service Connections’ was formed by contributions from several participants who highlighted the need for knowledge of services beyond primary care. Value was placed on accessibility of contacts and services in secondary care. Participants who were employed in a split role between primary and secondary care and were familiar with the processes, pathways and contacts in each clinical environment emphasised this advantage.

In my trust, our band sevens are normally a blended role; it's good because it means that the system is very joined up. So, when I refer into orthopaedics, I know those people, I can talk to them about what I need to do to refer, or is this appropriate? It's all very joined up, which I think is often not the case when people just work in FCP because they're a bit sort of marooned.

(P8)

Participants who were only employed in primary care explored the complexities of developing these service connections and found that a small number had managed to access time in secondary care as part of their FCP clinical learning experience.

…. the value of observing practitioners in secondary as well as primary care, because I suppose that's half the job; referring on and knowing if you're referring on appropriately, things to look out for, that kind of thing.

(P5)

Resource availability was the second category of this theme; variation was found between participants. Some were provided with accessible clinical supervision in their own place of work, the organisation was supported by service colleagues and time was allocated.

The PCN were aware that we were both doing the Roadmap…so when it became obvious that we'd blocked an afternoon to do it together, there were no questions asked. They were supportive.

(P10)

Others reported challenges in accessing the resources required to complete this compulsory activity. Whilst accepting personal responsibility for scheduling supervision, some had to address contractual considerations. A participant employed by an NHS Trust and then working for part of their role in primary care explored this complexity:

The PCN clinical leads felt that the element of supervision required formally should fall on the time of the hospital Trust; they were employing the Trust to provide a FCP service and felt that it was the time and responsibility of the hospital Trust to provide that supervision and not for them.

(P4)

Another explained that their employer support was restricted and contractual complexities resulted in them completing the clinical supervision in their own time and unpaid.

It wasn't part of my normal working hours, so it was in addition to my normal work. From a financial point of view as well, it was something that I had to do myself; it wasn't paid work.

(P9)

### 3.3 Theme: Role of Personal and Professional Identity

The volume of material related to individual participant's personal characteristics and the relevance of these was noteworthy. Participants reported that, as clinicians, their own cultural background, personal and professional identity influenced their clinical role and patient interactions.

I was based in a very rural, middle to upper class, white, Caucasian area; a small village. Everyone's very well‐to‐do. So I was the only person of colour there… Understanding of different cultures isn’t there yet, which can act as a barrier.

(P2)

A participant who had trained as a physiotherapist outside the UK explained how their lack of familiarity with the UK system presented some specific learning needs.

I sat in with a lot of people because when I started, I was only four months into the FCP role, so I really wanted to understand the system, because again, I'm not from the system. I didn't grow up in the system.

(P3)

Some participants extended the discussion to include how their clinical learning needed to acknowledge and be responsive to patients' individual features, including their cultural backgrounds.

My FCP mentor did point out a couple of bits about ethnic minority patients, which were useful. I think they were in the back of my mind. Indian patients having a higher prevalence for diabetes, for example.

(P1)

Participants raised the significance of neurodiversity both for themselves as students but also the need to identify and acknowledge relevant neurodiversity issues with patients. As a dyslexic, one participant highlighted that the university route to meeting the RTP's requirements provided more guidance and structure to alternative routes.

Being dyslexic and also struggling with …. what I was doing, …. I thought I just need to go down the university route and get guidance on how to do the task.

(P12)

Others commented that in relation to patients' neurodiversity needs, formal training was limited but as clinicians in primary care, they wanted to offer individualised and responsive patient consultations.

In response to neurodiversity; trying to make reasonable adjustments, trying to ensure that contact with that patient is approachable and as beneficial for them as can be.

(P7)

Professional identity was a topic that participants drew on as they explored settling into and developing the FCP role. Participants reported a widespread lack of role understanding, commenting that they were regularly confused for ‘in‐house’ physiotherapists offering treatment and rehabilitation and several highlighted that many patient consultations were not first contact. The opportunities linked to this relatively new role were discussed by some, who were keen to highlight innovation, good practice and the impact of FCPs they respected. Topics explored included the FCP's ‘*position in the team*’, (P7), the value of professional specialisation in primary care ‘*how much I love being a (musculoskeletal) physio and not having the pressure* (of other specialisms)’, (P2) and the potential to ‘*make your own path as an FCP*’, (P8). As participants explored their professional identity, they made references to the scope of practice and associated complexities. Several noted that whilst additional skills could be acquired and brought into the FCP clinic e.g. injection therapy and independent prescribing, navigating skill integration had to be carefully considered.

My GP is really supportive of me increasing scope of practice and being able to do that. There's always that potential fear of ‘treading on toes,’ and not taking away from what the GP's do. There's a lot in the world of social media about the role of physician associates and what their scope of practice is. As a physio, you feel where does that scope of practice start and end? Where does it cross over into GP territory?

(P4)

The nuances of professional identification as a musculoskeletal FCP were noted by all; individual, service wide and national considerations were reported with a consensus that role identity was not interpreted in a standard way. One participant had used the university's coursework to explore this topic in detail:

I did my service evaluation piece (module assessment) on the understanding of our role, our professional identity.

(P11)

### 3.4 Theme: Dynamics of Learning

Participants all discussed factors relating to the dynamics of learning, theme categories were: impact of directed learning, influence of mentorship relationship, effectiveness of mentorship and bidirectional learning. Participants reflected strong and positive opinions relating to the value of clear direction and guidance provided by the university.

…everything was very clear about what was expected.

(P3)

Two commonly reported findings related to the clinical mentor's role and impact. Participants reflected positively that the clinical mentor did not complete the formal module assessment—this was the university's responsibility. The clinical mentor's role was to facilitate clinical reasoning, reflection and learning in the clinical environment:

It was a positive in the fact that their focus wasn’t on the marking side of things, it was more on giving me that feedback on practice only.

(P7)

I think that my mentor not grading me probably made me more relaxed in terms of being able to have open discussions, and being more of a learning process, rather than an assessment process.

(P5)

The second commonly reported opinion was the value of accessing more than one mentor and the acknowledgement that different individuals will support different practice elements.

I draw on experience of a variety of members of the primary care team and also secondary care as well. So, I had a good, varied experience with different professionals.

(P1)

Accessing professionally diverse team members throughout the learning experience was regarded positively:

I sat in with as many people as I could…… they were really good. The pharmacists and the wellbeing coach and the social prescribers, they were very on board and understanding.

(P11)

So I suppose we've all got different strings to our bow and things that we’re good at. Someone supervised me who was a very good communicator; very empathetic approach, shared decision making. So I learnt a lot from her and then another person is really into green prescribing, I learnt a lot about local resources and projects.

(P5)

Participants reflected on their clinical learning and shared positive experiences that they felt had enhanced their practice.

The GP's were very refined and to the point with their agenda setting and gaining the patients ideas and expectations specifically, so I think that fed in really nicely to my own practice.

(P7)

Reducing and making my history maybe a little bit more concise, making my questioning a bit more concise. Talking less.

(P11)

So, there was a lot of chronic pain. There was a lot of cases of mental health issues and people with financial issues.

(P9)

A small number of negative experiences were shared when participants did not feel a link between their personal development needs and the mentorship.

There were a few times when he did jump in and start talking… and it becomes a dialogue between the patient and the person observing.

(P10)

Several participants explored the impact of the mentorship activity beyond their own learning noting mentors appeared to have benefited from the experience. For some, it had been an opportunity for the development of professional relationships as mentors and mentees had learnt from each other.

He said I was the first person that really challenged him, but that's a good thing.

(P2)

My mentor was incredibly supportive of the FCP role and very much recognised the value of having an expert musculoskeletal opinion within the clinic and wanted to learn from me as well.

(P4)

Participants made links between the lack of formal assessment undertaken by the mentor and patients' perceptions of the process. Reflecting on discussions had with mentors and the fact that bidirectional learning sometimes took place, the patients' reactions were generally positive:

I'd have discussions in front of patients with my mentor, the patients were always happy and consented, and they quite enjoyed being talked about.

(P6)

I never got the impression that people thought that I was being mentored, it was more just a collaborative approach. I introduced it (supervision) as a routine thing, that everyone should be doing as part of normal practice.

(P5)

## 4 Discussion

This study aimed to understand key elements of clinical supervision, explore associated facilitators and barriers and support the development of supervision models for FCP's. These will be discussed alongside the three themes emerging from the data.

### 4.1 Operational Factors

#### 4.1.1 Category—Service Connections

This review highlights the challenges of FCPs with navigating referral systems across both primary and secondary care. The role of FCP's as gatekeepers for secondary care referral has been a key aim of FCP provision (Goodwin et al. 2021; Greenhalgh et al. 2020; CSP, 2018). The CSP (2018) has previously documented that FCPs are better integrated across musculoskeletal pathways with dual roles in primary and secondary care than those who are employed by the incumbent musculoskeletal provider. Being able to identify and streamline appropriate patients quickly and efficiently is seen as pivotal for practicing clinicians (NHS England 2019). A lack of clear referral pathways has been highlighted previously (Baird et al. 2022) and FCP's working within ARRS roles may struggle to navigate the healthcare system if clear referral pathways to secondary or specialised care are not established. This study highlights the requirement for stakeholders in clinical practice to enable and operationalise appropriate protocols and policies. These should enable FCP's to escalate cases or refer patients to specialists when needed.

#### 4.1.2 Category—Resource Availability

This review highlighted that whilst supervision is an essential component of the RTP to ensure safe and effective patient care, clinicians faced challenges accessing supervision and aligned with other studies (Baird et al. 2022; Nozedar and O’Shea 2023; Ingram et al. 2023). Barriers including limited supervisors/mentors, time constraints, unclear expectations, workload and resource strain have been identified as previous obstacles (Baird et al. 2022). Time constraints and pressures are major factors contributing to supervision difficulties in busy primary care settings (Baird et al. 2022; Nozedar and O’Shea 2023). Having access to regular, high‐quality supervision maybe challenging for clinicians juggling clinical and administrative responsibilities, but the need for non‐patient contact time has been recommended (Nozedar and O’Shea 2023).

The organisation and procurement of supervision is likely to be underpinned by stakeholder relationships, funding arrangements and administrative support. This review highlighted a lack of organisation and contractual impacts as barriers. FCP's and their supervisors are likely to require administrative support to ensure that supervision is properly documented and feedback provided. Without support, the supervision process may become cumbersome or neglected, perpetuating clinical uncertainty, feelings of stress, and clinical burnout (Ingram et al. 2023; Danczak and Lea 2017). Contractual arrangements without supervisory detail or funding agreements within current ARRs roles have been highlighted by previous reviews (MacConnachie 2024; Baird et al. 2022; Bramwell et al. 2024; Millington et al. 2024). Funding to cover time required for supervision sessions, and additional administrative support should be considered by stakeholders in future provision.

Additional implications for clinical practice relate to acknowledging the need for psychological and operational support relating to supervision. Enabling FCP wellbeing by ensuring support is available for these clinicians who may experience elevated levels of stress, burnout or fatigue (Nozedar and O’Shea 2023; Thompson et al. 2024; Welford 2018; Ingram et al. 2023; Venturini et al. 2024) is a consideration of current and future stakeholders.

### 4.2 Role of Personal and Professional Identity

#### 4.2.1 Category: Equality, Diversity and Inclusion

The cultural backgrounds of clinicians and patients were identified in this research as impacting learning and practice. Challenges of FCPs working with ethnically diverse populations and the impact of health beliefs have been previously identified by Greenhalgh et al. (2020) leading to recommendations for cultural competence training. Addressing cultural competence more fully within FCP teaching, clinical supervision, and in future iterations of the RTP is recommended and could significantly improve FCP job satisfaction, patient outcomes and experiences (Kumar et al. 2019; Oelke et al. 2013).

The impact and challenges of neurodiversity were raised both from a student and patient perspective. The challenges and positive aspects of neurodiversity for healthcare clinicians have been well documented (Butler 2024; Crouch 2019). With the completion of the RTP being essential for an FCP role, this research suggests that neurodiverse students' needs may be best met in a HEI taught route. The collaborative learning environment created by studying with others offers belonging, support, community, diversity of thinking, understanding and emotional support that can assist with confidence for neurodiverse clinicians (Butler 2024; Robinson 2022). Future modifications to the RTP content and options for completion should consider neurodiversity more fully to improve accessibility.

Whilst challenges of access to healthcare for people with learning disabilities are well recognised (Alborz et al. 2005), there is a desire from FCP clinicians to be responsive to the needs of individual patient's neurodiversity needs. The introduction of The Oliver McGowan Mandatory Training on Learning Disability and Autism (NHS England (b), n.d.) may have raised awareness of this topic, but its effectiveness or impact on patient experience has not been measured. Gaps in the current evidence‐base present an opportunity for future high‐quality research related to delivering patient‐centred care for all (HEE/NHSE 2017).

#### 4.2.2 Category: Exploration of Professional Identity of FCP

Professional identity concerns were highlighted in the early stages of the development of the FCP role (Greenhalgh et al. 2020; Morris et al. 2021, Wood et al. 2022) and have been identified by other professional groups integrating into primary care (Jorgenson et al. 2014). Nearly 5 years after the launch of the RTP, professional identity remains a key theme highlighted by recent research (Ingram et al. 2023; Lewis and Gill 2023; Lamb et al. 2024; Thompson et al. 2024) and by all participants in this study with a lack of understanding about the FCP role remaining, and many still being confused for ‘in‐house’ physiotherapists. The benefits of the FCP role for patients are clear (Wood et al. 2022), but lack of role understanding may affect patient accessibility to this service, prevent effective utilisation of the extended scope skills many of these clinicians offer and potentially impact clinical supervision strategies.

The speedy roll out of the role, constraints to support offered from overseeing organisations and the relatively novel nature of physiotherapists being in Primary Care could all contribute to the lack of understanding of the FCP role, but further research and role promotion support is urgently required to maximise the positive impact of this role.

### 4.3 Dynamics of Learning

#### 4.3.1 Category—Impact of Directed Learning

Findings from this review demonstrate the value of having clear direction and guidance provided by a university course. Having a full understanding of what training and supervision is required in a new role is fundamental to the learning process (NHS England 2021). RTP documents aimed to establish the capabilities of Allied Health Professions (AHPs) moving into primary care and identify the supervisory support required via either a portfolio or taught university route. However, these roadmaps have been highlighted as being confusing and unclear (Jones et al. 2024) and consequently seen as a barrier to completing the portfolio route. The high level of satisfaction with university support identified in this review aligns with previous research identifying the taught route as being a more attractive option (Carus et al. 2023).

Findings from this review revealed universal support for formal assessment to be carried out by the university and not the clinical mentor. One supervision element is the completion of paperwork, which is not only time consuming but can also be difficult due to complex marking criteria (Carus et al. 2023). Taking away this responsibility protects the mentors' time whilst valuing their contribution to learning through observation, open discussion, and critical feedback. It also challenges the hierarchical relationship of traditional mentoring and allows a more collaborative approach (Teo et al. 2024).

#### 4.3.2 Categories—Influence of Mentorship Relationship and Effectiveness of Mentorship

Another aspect of learning emphasised in this study is the benefit of accessing more than one mentor from a range of professions to support different elements of practice. In recent years, it has become increasingly common for supervision to be conducted across disciplines, not just for FCPs but for all medical professionals (Launer 2018; NHS England 2021; McGuinness and Guerin 2024). Some of the benefits include offering different perspectives to practice (Launer 2018) and providing a catalyst for new ways of working (Davys et al. 2021). This may offer considerable benefits to clinicians such as FCPs, who are moving into new roles and settings (Millington et al. 2024). In addition, previous literature has acknowledged ‘supervision fatigue’ with GPs often supervising medical students, foundation doctors, and GP registrars as well as ARRS staff within a practice (NHS East & West Midlands Clinical Senates 2025). Therefore, taking a multi‐professional approach to mentoring and supervision is also an effective way of sharing this responsibility.

#### 4.3.3 Category—Bidirectional Learning

Clinical mentorship is increasingly recognised as a bidirectional process that benefits both mentors and mentees (Burgess et al. 2018). Having FCPs with valuable musculoskeletal expertise has been highlighted as an opportunity for other members of the primary care team to upskill in musculoskeletal care (Moffatt et al. 2018), and this review further establishes this beneficial relationship. Given the consistent lack of understanding of the role and scope of FCPs (Goodwin et al. 2021; Lamb et al. 2023), this bidirectional learning also offers an opportunity for other healthcare professionals to gain greater insight and ensure the full potential of FCPs can be realised.

## 5 Strengths and Limitations

This study methodology has been optimised through alignment of its key domains: research team and reflexivity, study design, analysis and findings with the COREQ consolidated criteria for reporting qualitative research (Tong et al. 2007). Researchers' reflexivity has been considered; their established understanding of the musculoskeletal FCP role has enabled participant recruitment, high level discussion during interviews, and analysis that was subsequently explored with reference to the contemporary healthcare landscape. Engagement of participants in data checking, analysis and the publication process has further contributed to methodological rigour (Stringer and Aragón 2020). Potential bias of the research team must be considered as they were all employed at the university where the participants had completed their FCP training but the strategies outlined above were used to ensure that transparency and credibility have been optimised. Recommendations for future research have been proposed in Table 3.

**TABLE 3:** Suggestions for future research.

Exploration of referral patterns and clinical outcomes of FCPs from varying employment frameworks e.g those employed directly by primary care service and those from integrated services
Evaluation of supervisory models with more than one profession/professional involved
How do clinical supervisors and higher education institutions address trainee and patients' individualism for example, cultural diversity and neurodiversity during clinical supervision?
Exploration of service provision leads' and policy makers' understanding and responses to financial, resource and contractual elements associated with FCP supervision
What is the clinical training experience for FCP supervisors? Is bidirectional learning a consistent outcome?

## 6 Conclusion

Clinical supervision is a requirement of FCP role training that presents an opportunity for professional development. Its success and impact are dependent on governance structures that support mentor availability and enable engagement with a range of healthcare professionals. The FCP role is not consistently interpreted, and individual clinicians have acknowledged that their personal and professional identities are evolving; factors such as cultural competence and further training relating to neurodiversity should be considered as part of FCP training. Integration of pedagogical principles relating to optimal learning dynamics should be considered when designing and implementing FCP clinical supervision.

## Author Contributions

**Sue Innes:** conceptualisation, pilot interview, data curation, formal analysis, project administration, writing – original draft, writing – review and editing. **Sarah Golding:** ethics application, interviewer, methodology, writing – original draft, review and editing. **Philip Nardone:** data curation. **Caroline Kerry:** pilot interview, writing – original draft. **Anthony Smith:** writing – original draft. **Sarah‐Jane King:** writing – review and editing. **Fiona Blackman:** data review, and writing the original draft.

## Conflicts of Interest

The authors declare no conflicts of interest.

## Data Availability

The data that support the findings of this study are available on request from the corresponding author. The data are not publicly available due to privacy or ethical restrictions.

## Appendix 1

### Interview Topic Guide

#### Introduction/Consent

Welcome to this interview and thank you for agreeing to take part in our research study. Today is *[insert the date and time of the interview]*. As you are aware, we are exploring FCP experience of supervision. The participant information form has been provided and summarises the topics that will be investigated; we hope to explore these in this interview. Are you happy with the information which you have received about the study and do you understand this fully?

Do you agree to participate in the research project, ‘First Contact Physiotherapists experiences and reflections of the clinical supervision element of part 2 of the “Roadmap to Practice” (Higher Education Route)’ and for me to record this interview?

### Interview Questions

1. First of all, thinking back to your stage 2 FCP supervision, can you clarify when this took place?
2. Are you able to reflect on your supervision, can you summarise the key elements, for instance who was involved, how did you feel it went, any positive or negative experiences?
3. Clarification of professionals involved (FCP or GP). What proportion involved which professional?
4. Reflecting on the different clinicians, what was similar and what was different?
5. What do you think constitutes good practice within supervision? You have already mentioned (e.g. time, approach), can you expand?
6. Was there anything about how the formalities or structure of the supervision and arrangement affected the power dynamic between you and the supervisor? (for this specific stage 2 part of the ‘roadmap’ the supervisor didn't have to formally assess/judge you, did this have any influence on the supervision dynamic?)
7. When you reflect back on the supervision period, were there any limitations to your supervision—anything that you feel could have been enhanced?
8. If multi‐professional supervision: You had supervisors with different professional roles that is, a GP and an FCP, was there anything that you received which was different from the individual supervisors within their different professional roles? How did this impact your professional development?
  OR: If single profession supervision: You had supervision from 1 profession, what was the impact of this? Can you consider how things may have differed if you had accessed supervision from more than 1 profession?
9. What was the effect of the whole supervision process and supervisors' profession(s) on your personal professional identity? (Explore understanding of scope). Did your view of your role as an MSK Physiotherapist change? Did this affect your approach to MSK consultations? Was there anything you changed in clinical practice as a result?
10. Thinking now of the individuals involved, yourself and patients, how responsive was the process to all individuals' features and needs? Prompts relating to equality, diversity, inclusivity, health literacy, socioeconomic factors, learning needs and neurodiversity.
11. Was your supervision conducted locally with your own patient population/caseload or elsewhere? What was the impact?
12. Considering the coursework completed for this part of the FCP Roadmap, what was your view of the assessment methods and your personal circumstances?
13. Is there anything else you would like to add? Thank you very much for your time.

#### Closing

We will transcribe and analyse the data once all the interviews have been conducted. We are inviting participants to take part in data verification analysis and elements of the publication writing up. Is this something you would want to be contacted about?
